# Dr. Prout on the Nature and Treatment of Gravel, Calculus, &c.

**Published:** 1821-06-01

**Authors:** 


*An Inquiry into the Nature and Treatment of Gravel, Calculus, and other Diseases connected with a Deranged Operation of the Urinary Organs.* By William Prout, M.D. F.R.S. London, 1821, pp. 227.

In the course of our critical avocations, it has seldom occurred to us to rise from the perusal of a medical work with such favourable impressions as in the present instance. It is too often our lot to have our attention caught by glaring marks of hurried composition, and of inaccurate reasoning, or to find the author copying whole passages from preceding writers, without any attempt at verifying their observations. Dr. Prout is an author of a very different stamp, and to understand his work, it must be examined in a very different way from that which may frequently be practised with modern medical productions, without any injustice to the author. We mean to say, that it is not here sufficient to turn over the pages, and occasionally to pause upon a particular passage. This is a volume which must be accurately studied to be thoroughly understood. The author tells us that, for many years past, he has been in the habit of attending closely to the diseases of the urinary organs; and from the whole tenor of the work it is clear that he has done so, and that his pathological views have been almost exclusively the results of his own *individual* observation. An air of *originality* certainly prevails throughout this volume, which is highly creditable to the author, and the feeling of which was certainly predominant with us while engaged in its perusal; but we are far from saying, that it does not come recommended to us by other and higher merits than the mere novelty of the views which it opens. It is the work of an *accurate* as well as of an *original* writer. Dr. Prout has laid the best possible foundation for becoming a good pathologist, by the assiduous attention which he has long paid to the subject of animal chemistry in general. We do not now propose to discuss the question *how far* the doctrines of animal chemistry may be made subservient to the promotion of physiology and pathology, but of this we are sure, that accurate notions in that branch of science must prove at all times a most useful check on physiological and pathological speculations; and that, without them, an author is continually exposing himself to the risk of error.

It would be injustice to the writer not to add, that he has another merit almost as great as that of originality and accuracy. He is a very *cautious* pathologist. Conscious of the extent of his subject, and of the necessity of extensive and varied observation for its full elucidation, he confines himself to the illustration of certain positions, and makes no attempt to anticipate, by a bold hypothesis, the labours and honours of succeeding authors. The subject which he has chosen may not, at first sight, appear calculated to display, to much advantage, the peculiar talent which we are disposed to give this author credit for. The diseases of the urinary organs are few, and, comparatively speaking, of rare occurrence; and the interest which attaches to them is therefore certainly less than to many other subjects in pathology; but we shall find that Dr. Prout's views have an application far more extended than could have been anticipated from the professed object of the work. They have a bearing upon *general disease*, as much as upon disease more immediately connected with a deranged operation of the urinary organs; and this we would urge as a most important feature in the character of the work which we are now to analyze.

After expressing ourselves in this manner of the author, and of the general value of his book, it may appear like presumption in us to differ from him; but with the same freedom with which we mention the merits, we shall speak also of what appear to us to be the faults of the work; and in this spirit, for which we have the author's own assurance that he will thank us, we enter upon our pleasing task.

The introduction presents us with a contrasted view of the elementary principles of the blood and urine in man. In this portion of the volume we do not observe any thing deserving particular notice, if we except, perhaps, a hint thrown out, (page 22) that the colouring principle of the urine may possibly be of two distinct kinds. One of these has an affinity for the lithate of ammonia, and a peculiar tint, and is that to which the lithic acid calculi appear chiefly to owe their colour. The other has no affinity for lithate of ammonia, is ill defined, and of uncertain composition; and yet in some manner it would appear to be intimately connected with the former as well as with lithic acid. The general results of this examination of the principles of the blood and urine may be best given in the author's own words.

"From the preceding sketch, we find that the most striking differences between the blood and the urine, is the complicated nature of the latter. The astonishing variety of substances formed from such a paucity of materials, naturally leads us to reflect upon the vast extent of the operation of the kidneys. On considering, however, a little more attentively the nature of the operations of these organs, we shall find, as Berzelius has justly remarked, that *acidification* constitutes the chief feature in them. Thus, the sulphur and phoshorus of the blood are converted by the kidneys into sulphuric and phosphoric acids: a new acid, the lithic, is generated altogether, &c. Such, then, evidently is the natural and healthy operation of these glands. We find, however, that in certain forms of disease, this acidifying tendency is carried to excess, and nitric acid, oxalic acid, &c. are produced. On the other hand, it is occasionally suspended, diminished, or altogether subverted; and unchanged blood, or albuminous matter; neutral substances, as urea, or sugar; or even alkaline substances, as ammonia, lime, and magnesia, are separated in abundance; and the phosphorus and sulphur at the same time pass through the kidneys without being acidified. With respect to the character of the diseases attending these states of the urine, it will be generally found, that when acids are generated in excess, the urine is commonly small in quantity, and high coloured, and the disease inflammatory: when neutral or alkaline substances, the urine, on the contrary, is generally pale coloured, and larger in quantity; and the diseases are those of irritation and debility." P. 31.

After acknowledging the difficulty which exists, in the present state of our knowledge, of devising an unexceptionable mode of classing the diseases connected with morbid states of the urine, the author assumes as the basis of his arrangement the simple principle of the *solubility* or *insolubility* of the principles met with in urine. He divides his subject accordingly into two sections; the first including *diseases in which principles soluble in the urine are morbidly deranged in quantity or quality;* the second, *diseases in which principles insoluble in that secretion are similarly deranged.* Under the first of these general divisions he arranges—1. Various forms of albuminous urine. 2. Anonymous diseases, in which the characteristic symptom is an excess of urea. 3. Diabetes. Under the second, all the various forms of gravel and calculus. As a general principle of primary importance in all diseases, it is then urged that the mere circumstance of a *diminution* or *increase* in the flow of urine will seldom fail to furnish us with a clue to the actual condition of the system, and consequently to the principles upon which a disease is to be treated. A

diminished flow of urine accompanies an inflammatory state of the general system. An increased flow of urine, (or diuresis) very constantly accompanies the state of nervous irritability. As we proceed in the analysis of the work, it will naturally occur to our readers that there is more science in the system of the professed *water-doctors* than the world has yet had the charity to allow them.

These remarks are introductory to the consideration of *albuminous urine*, with which the author sets out. That urine often contains a large quantity of coagulating matter, albumen, has been long known, and the fact was lately urged upon the attention of practitioners by Dr. Blackall, as one of great importance in the diagnosis and treatment of dropsies. It has hitherto been always imagined that the urine is rendered albuminous by the serum of the blood. Dr. Prout, however, is strongly inclined to believe, that it is derived immediately from the chyle. He has observed that the albumen of the chyle differs in its chemical characters, as well as in its sensible properties, from that of the blood; and that the albumen found in urine is most generally similar to the former. The occasional presence of chyle in the urine is the chief novelty in the pathological views which the author gives us in this first chapter; but there are several interesting particulars here brought forward regarding the albuminous condition of the urine, and the states of the system in which it is observed, which it is also necessary to advert to.

In almost all cases of albuminous urine, the usual principles found in this secretion, (urea, lithic acid, &c.) are deficient in quantity. Hence albuminous urine scarcely ever exhibits a pink sediment. Dr. Wells, indeed, observed two instances of such an occurrence; but it is undoubtedly very rare. Urine which is passed some length of time after a meal is always the most loaded with albumen. One instance is recorded, (page 41,) where the urine, passed in the evening, after an early dinner, became, on cooling, a solid coagulum, assuming the shape of the vessel, and resembling blanc-mange. This albuminous condition of the urine occasionally exists in the most healthy, and often unsuspected. The constitutional symptoms which attend it are seldom severe, and may be characterized as unpleasant rather than dangerous. In a case recorded by Dr. Wells, the disease continued for nine years. When such a state of the urine is *permanent*, and *excessive*, it necessarily indicates some great derangement of the animal economy. The symptoms attending it are stated to be as follows —

"In slighter cases there is generally a frequent desire to pass water, and for the most part decided diuresis. I have never known albuminous urine attended by positive pain, though the patient, for the most part, complains of certain indescribable sensations, which render him conscious that all is not right. In severe cases, where the drainage from the system is greater than natural, there are, as might be expected, an inordinate craving for food, and other symptoms somewhat resembling diabetes." 39.

Such a condition of the urine appears to be excited by a variety of causes, though in many instances its origin cannot be ascertained. The author has observed it to follow a long course of mercury, the use of stimulating diuretics, exposure to cold, and violent passions of the mind.

The author evidently distrusts Dr. Blackall's views of the pathology of albuminous urine, or rather, we should say, Dr. Wells's, for we are informed (page 48) that the notion of albuminous urine being connected "*with two great action in some part of the system*," originated with that author; yet with his characteristic caution, he refrains from giving a decided opinion on the subject. He does not deny that there may be cases where the urine is albuminous, in which blood-letting is useful, but he questions very much the propriety of the practice, whether in dropsy or any other disease, upon the *mere* ground of the urine being albuminous. That irritable state of the whole system, and especially of the kidneys, which usually attends the separation of albuminous urine, appears to the author to indicate the use of sedatives, and especially of opium; but he qualifies this afterwards by saying that albuminous urine does not, in the present state of our knowledge, lead to any particular remedy or mode of treatment. Now we may as well take this opportunity of observing that Dr. Prout appears to us to have an overweaning fondness for this principle of irritability, and its usual result, the general practice of exhibiting opiates, for which we know not how to account. This seems to us, indeed, to be the leading error of the author. We shall have occasion to find him alledging the existence of the same condition of the system, and recommending a similar practice in almost every disease of which he treats. We cannot persuade ourselves that he is always borne out in this by the symptoms, even upon his own shewing. In the case of *albuminous urine*, for instance, the only symptoms noticed are a frequent desire to pass water, and a degree of inordinate appetite. Yet these are at once declared (page 39) to be *symptoms of irritability*, and to *indicate the use of sedatives*.

Chap. 2. treats of those diseases in which an excess of urea is the characteristic symptom. As they have never yet received any name, nor excited any attention among pathologists, we think it may be advisable to point out in what manner this symptom is to be ascertained, and judged of, in order to direct the attention of our readers towards it in any obscure case which may come under their notice.

A little urine is to be put into a watch-glass, and we are to add to it carefully nearly an equal quantity of pure nitric acid, which, from its greater specific gravity, will subside to the lower part of the glass, allowing the urine to float above it. In healthy urine, the acid and the urea do not act upon each other until the urine is concentrated by evaporation. In certain diseased states of the urine, however, this occurs without evaporation, or what the author calls *spontaneously*, and a crystaline compound results. This happens where the urea is in excess. "The difference *(degree)* of excess can be inferred, near enough for practical purposes, by the greater or less time which elapses before the crystalization takes place, which may vary from a few minutes to two or three hours." (Page 11.)

The diseases in which an excess of urea may be considered in some degree as characteristic, have been sometimes denominated diabetes insipidus. They differ however considerably from genuine diabetes, though the *quantity of urine* voided is for the most part above the natural standard of health. The average specific gravity of the urine in these complaints may be stated at 1.020; most generally it is pale, but in some instances it has been observed high-coloured, resembling porter, more or less diluted with water. It is very prone to decomposition, becoming speedily alkaline, especially in warm weather.

The principal symptom of the affection thus characterized is a frequent and irresistible desire of passing water, both by night and day. There is occasionally a sense of weight or dull pain in the back, with irritation about the neck of the bladder, extending in some cases along the urethra. But the negative symptoms of this anonymous disease are the most interesting. The functions of the skin are unimpaired. The pulse is not affected. There is no thirst nor craving for food, (except in severe cases.) The tongue is clean. The bowels regular. It has chiefly occurred to the author to observe it in persons free from gout, or any organic defect in the urinary organs. In every instance they applied for medical advice from the dread of its ending in something worse, rather than from actual suffering. The causes of this particular state of the urine are unknown. The author conjectures that whatever debilitates may lead to it. He has had no opportunity of ascertaining the progress of the disease, but he thinks it extremely probable that some may terminate in diabetes, and others in a deposition of the earthy phosphates. He recommends sedatives, and particularly opium, as "the only efficient remedies;" but by these he considers it probable that the disease may, in most instances, be suspended, if not altogether removed.

The third chapter treats of the very interesting subject of diabetes. We fully concur with the author in the necessity of confining this term to those affections in which the urine is saccharine. A vast number of diseases are accompanied by a large flow of urine. Some of these are of a temporary and local nature; others are more permanent; and if these are all to be called *diabetes*, we may despair of ever improving the pathology or treatment of the formidable disease to which that term does certainly more strictly apply. We do not think there is any thing very new in the author's observations concerning diabetes, but it is interesting and instructive to view this disease in conjunction with others with which it is liable to be confounded—to view it as one of a series, and not as that isolated complaint, in which light, as it appears to us, pathologists have hitherto been contented to view it.

The author is indebted to Dr. Watt, of Glasgow, for the detail of symptoms ; and to Dr. Henry, of Manchester, for a very useful table pointing out the quantity of solid extract contained in urine of given specific gravities. To shew the value of this table, we may mention that we are at present in attendance on a diabetic patient, who passes ten pints of urine per day of the specific gravity 1.039, as determined by Mr. Faraday. By consulting this table, we perceive that he voids sixteen ounces of solid matter in twenty-four hours.

The observation of this case enables us to point out one peculiarity in the symptoms of diabetes, which Dr. Prout overlooks. He tells us that the taste of the urine is always decidedly saccharine, in a greater or less degree. Now in the case just mentioned, the saccharine taste was so obscured by urea and the saline principles (always present in diabetic urine, though in diminished proportion) as not to be perceptible until the urine was concentrated to a considerable degree by evaporation.

Diabetes has been known, according to the author, to terminate in three ways; the first and most common is by pulmonic symptoms, accompanied by hectic fever; the second is by dropsy; while, in a few cases, the patient has been cut off suddenly. In investigating the pathology of diabetes, the first question that occurs is, whether the increase in the *quantity* of the urine is owing to its saccharine condition, or is dependent on some other cause. The author inclines to the idea that it is independent of the change of quality in the urine, and probably referable "to that irritable state of the system which forms a part of the disease, and which resembles that peculiar condition sometimes present in hysteria and other nervous affections, in which a large flow of limpid urine frequently takes place." (Page 65.) Some of the constitutional symptoms attendant on diabetes are, perhaps, owing to the saccharine condition of the urine, but many of the most distressing are more probably referable to that enormous drainage from the system, both of solid and fluid matter, which takes place when the disease is severe.

The distinction between the increase in quantity and the alteration of quality in the urinary secretion occurring in diabetes, is stated (page 67) to be very important with reference to the operation of remedies. Animal diet affects (diminishes) the quantity, but we are not authorized to say, from observations yet made, that it improves the quality of the urine. "Blood-letting stands precisely in the same predicament." "I think, however," adds the author, "that there are stronger grounds for presuming that blood-letting has improved the quality of the ur ne, than that animal diet has produced this change."

Long before Dr. Prout had written upon, or, perhaps, directed much of his attention to, the disordered conditions of the urinary secretion, opium had been recommended for the cure of diabetes; and this is considered by the author as affording some countenance to his view of the disease. He tells us (page 76) that "we have abundant proofs of the power which opium possesses of effecting changes in the urine which appear to be of a curative nature." He agrees with a late writer on the subject, Dr. Elliotson, in thinking that the three remedies, animal diet, blood-letting, and opium, do not interfere with each other; and he coincides in the recommendation of having recourse to them conjointly. Now really we are at a loss to understand this system of blowing hot and cold with the same breath. That bleeding may be adapted for one patient, and animal diet for another, we can readily believe; nay, we shall find no difficulty in seeing how they may be employed with advantage in the same individual in different stages of the complaint; but that they can be mutually serviceable at the same instant of time, is what we cannot comprehend. If they can be made so in diabetes, we do not see why they should not be united with equal advantage in the treatment of pneumonia.

By way of conclusion to the first section of the work, the author points out the analogy subsisting between the three morbid conditions of the urine already noticed; and attempts to shew that the bond of connexion between them is a kind of *hysteric* irritability of the system. He thinks it probable that in some instances they gradually run into each other, or into a fourth state of disease closely allied to the same class, to which, in a strictly natural arrangement, he should next have proceeded, viz. *the deposition of the earthy phosphates.* For particular reasons, however, he separates their consideration; but as we are not tied down by the same necessity, and as our chief object is to illustrate principles of pathology, we shall here briefly notice the author's very ingenious ideas concerning the deposition of the phosphates. To use his own language, we shall sketch an outline of the *phosphatic diathesis.* (Chap. 5. Sect. 2.)

It consists in a suspended or diminished action of the usual acidifying powers of the kidney, whereby, instead of lithic acid, a greater quantity of urea (equivalent to ammonia) lime and magnesia is generated. This condition of the urine is very commonly dependent upon a deranged state of the chylopoietic viscera; frequently, too, it is connected with a great degree of *irritability* and *debility* of the system. Hence it is that children are so liable to this form of deposite, from their extreme irritability, and great tendency to disorders of the stomach and bowels.

The deposition of the phosphates is attended with uneasiness about the loins, a sallow, haggard countenance, black, clay-coloured, or yeasty stools, and subsequently great languor and debility, as in diabetes. The urine too here, as in the diseases already treated of, is pale, and secreted in larger quantity than natural, but it is commonly of very low specific gravity, such as 1.002. When the specific gravity is greater than this, the phosphatic sediment is proportionally more copious. In this state of disease the urine is very prone to decomposition. It speedily emits a nauseous smell from the evolution of ammonia. The relation between this state of disease and the three formerly noticed, is considered to be established by these points of analogy. But to understand the full scope of the author's views regarding the deposition of the phosphates, it is necessary for us to go back to the commencement of the second section, in which those diseases are treated of, characterized by derangement, either in point of quantity or quality, of principles *insoluble in the urine.*

The first chapter gives a general description of the mechanical deposites which take place from urine, either within or without the body. They are of three kinds:—1. The pulverulent or amorphous sediments; 2. Crystaline sediments, usually denominated gravel; and 3. Solid concretions, or calculi, formed by the aggregation of these sediments. A summary account is given of the varieties of urinary gravel and calculi, with their chemical composition, chiefly extracted from Dr. Marcet's excellent essay "On the Chemical History and Medical Treatment of Calculous Disorders;" but as our readers must have been made familiar with this branch of the enquiry by many recent publications, we pass it over in order to afford more space for subjects less generally known.

The second chapter is a very interesting one. It is occupied with data, derived from the examination of different collections, shewing the comparative prevalence of the different forms of urinary deposite, and the order of their succession. Upon these data are founded a series of observations illustrative of the general pathology of calculous disorders.

In common with all writers on this subject, Dr. Prout lays great stress on the universality of the lithic acid, either as the nucleus or as the body of the calculus; but he says, that it is by the examination of the *alternating calculi* that a true notion is to be formed of the pathology of this disease. He investigates the *modes of transition*, by observing the different layers in contact with each other, and appears to us to have arrived, in this way, at some very important results.

The transition from the formation of lithic acid to the deposition of the phosphates is most interesting and instructive. It takes place gradually through the lithate of ammonia, and is accompanied by the disappearance of the usual colouring principle from the urine. The transition from the mulberry calculus to the phosphates takes place through a mixture of oxalate and carbonate of lime. The next layers are found to consist of the carbonate and phosphate of lime, and still farther from the centre, the carbonate of lime disappears. The author is inclined to believe, that wherever the change takes place *ex abrupto*, it is presumable that some time must have elapsed between the deposition of the different matters. It is a curious and important feature in the pathology of the urinary system, that a *decided* deposition of the phosphates is never followed by a different deposite. The author evidently distrusts altogether the existence of what have been called compound calculi—that is, calculi where different ingredients are intimately mixed. A comparison of the different varieties of calculi shews that there are three primary and essential distinctions among them, and these the author next treats of under the denomination of the three forms of calculous diathesis. He calls them (rather quaintly to be sure) the lithic acid diathesis, the mulberry diathesis, and the phosphatic diathesis; and a separate chapter is allotted to the consideration of each.

*Lithic acid diathesis.*—Lithic acid is separated from the urine in two forms, amorphous and crystalized—in common language, as *sediment*, or as *gravel*. In the first form it is in combination with ammonia; in the second, pure. An excess of lithic acid in the urine arises from simple errors in diet, unusual exercise, or any debilitating circumstances. The speculations in which the author indulges, as to the manner in which these causes act, do not appear to us very satisfactory. The important principle seems to be this; that, if *imperfectly assimilated*, or *unnaturally albuminous* matter is brought to the kidney, it does, and must, in virtue of its natural action, convert such imperfect albumen into lithate of ammonia.

Dr. Prout divides the *sediments* of urine into three classes: the yellow, the red, and the pink; and attaches to this a much higher degree of importance than we are inclined to think it merits. The circumstances that chiefly struck us as worthy of notice are the following:—The yellow sediments consist of the lithate of ammonia tinged by the colouring principle of the urine. They are the sediments of health, or of the slighter forms of dyspepsia. Some persons are far more liable to them than others; and this denotes a tendency in such constitutions to an *excess of lithic acid and its consequences*. Red or lateritious sediments consist of the same matter tinged partly by the colouring principle of the urine, and partly by the *purpurates* of soda and ammonia. They occur in persons of a feverish irritable habit, and in such, are excitable by trifling causes. They may be observed also in gout, rheumatism, and hepatic affections. Pink sediments are characterized by being coloured *wholly* by the purpurate of ammonia. The presence of the purpurates is a never failing test of the presence of feverish or inflammatory action. Pink sediments accordingly occur in all inflammations, but they appear also in a very pure form in hectic fever, chronic liver disease, and frequently in dropsy. Sediments in the urine properly belong to the sweating stage of fever. They point out that fever has existed and is going off, rather than its actual presence.

The pathology of *gravel* next comes under consideration. After a good detail of the symptoms attending a fit of the gravel, the author proceeds to notice the circumstances under which crystalized deposites take place from the urine, and he observes that they are of two general descriptions, *natural* and *acquired*. The tendency to them is not unfrequently hereditary. "On the other hand, the disposition to generate these sediments in excess is, like gout, or rather simultaneously with gout, but too frequently acquired by indolent habits, and excess in eating and drinking." It is connected also with certain unknown causes, by which it becomes endemic in certain districts, "as for example, that of which Norwich may be considered as the centre, in which more calculous cases occur, than in the whole of Ireland or Scotland." Lastly, we are informed that renal calculi are sometimes owing to organic disease of the kidney, or parts connected with it. (Pages 130 and 183.)

After a few remarks on the prognosis in this form of disease, the treatment proper to be pursued, where a lithic acid diathesis prevails, comes under consideration. Errors in diet are of course to be chiefly guarded against, but we are warned that errors in *quantity* are of infinitely more importance than errors in *quality*. Where a strong disposition exists to the formation of gravel, alkalis are indicated, "*but they are seldom or never to be given alone*."

"To be really useful," adds our author, "they must be conjoined with alteratives and purgatives. The pil. submur. hydrarg. comp. or a pill composed of the pil. hydrarg. and antimonial powder, taken at night, and followed up the next morning with a solution of Rochelle salts and carb. of soda in a bitter infusion, may be had recourse to. A little of the same mixture may be taken two or three times a day, so as to keep the bowels fairly open; or, instead of this, a little magnesia may be taken in a glass of soda water, as often as it may be found necessary. This plan is to be persisted in for a considerable length of time, according to the severity and obstinacy of the symptoms; the alterative pill being gradually had recourse to at longer intervals, and the doses of the other medicines diminished in a corresponding manner." P. 140.

In a fit of the gravel we are directed to begin by the employment of febrifuge means, before recourse is had to stimulating diuretics; the author having seen great mischief done by a contrary system, "the sufferings of the patient aggravated, and even his life placed in extreme danger." (Page 141.) If the case is very obstinate, or suspected to be accompanied by some local disease of the kidney, we are advised to apply a galbanum plaster to the loins, or to insert an issue or seton.

The fourth chapter is chiefly dedicated to the *mulberry* diathesis. Our knowledge concerning it is small, but the author conjectures—1. That the formation of oxalate of lime by the kidney is connected with a distinct diathesis, *excluding the existence of other diatheses.* 2. That this mulberry diathesis is of the same *general nature* as the lithic acid diathesis. 3. That oxalic acid is here generated instead of lithic acid; and that it is actually secreted by the kidney, and not formed there, as some have conjectured, by the agency of nitric acid.

Calculi, composed of the cystic oxyd, are rare, but always exceedingly pure. Hence the author conjectures that the *cystic diathesis* is more exclusive than any other, and that it does not readily *pass* into any other.

We have already alluded to some of the author's ideas regarding the *phosphatic*, or earthy diathesis. On this subject he is certainly at variance with most of our appoved writers on calculus. One of the principal points of difference between him and them, (more particularly Dr. Marcet and Mr. Brande) seems to be this:—The latter have long taught us that a tendency to phosphatic deposition is given by a long course of alkaline medicine, and this opinion, we are quite satisfied, has done far more to sway the practice of the present day in gravellish complaints, than any other we could name. Now upon this Dr. Prout lays no stress at all. He allows that in a few cases it may occur, but as a general principle in the pathology of earthy depositions from the urine, he considers it of no importance whatever. The real causes of this state of disease are, he says, either local or general. A large proportion of the cases are owing to some injury of the back. It is an old observation, that such injuries produce alkaline urine. Excessive fatigue, severe and protracted debilitating passions, are among the other general causes of the affection. *Its principal local causes are irritations about the bladder or urethra, especially when operating for a considerable length of time.* This appears to be the leading feature in Dr. Prout's views of the phosphatic diathesis. It is certainly deserving of remark, that the same view of the subject had long ago been taken by Mr. Murray Forbes, who expressly states, that "when a foreign body gets into the bladder, *it would operate by irritation*, so as to occasion a redundancy of the phosphates."

Phosphatic deposites from the urine are sometimes amorphous, sometimes crystalized. One of the characteristics of this diathesis is, that the urine, on standing for a short time, exhibits the appearance of an iridescent pellicle, consisting of minute crystals of the triple phosphate.

In such a condition of the urine, the indications of cure are—1, to lessen the irritability of the system by narcotics, and especially by *opium*, in large and repeated doses; 2, to restore the general health by tonic, and other appropriate remedies. He particularly recommends the mineral acids, uva ursi, bark, and chalybeates. Purgatives, he says, should here be used *with great caution*, except in children, and those slighter cases where the strength of the system is not much diminished. This may be judged of, by the specific gravity of the urine being in such cases comparatively high.

> "In mild incipient cases, I have seen the greatest advantage from the combined use of the muriatic acid, hyosciamus, and uva ursi; conjoined with the use of alterative purgatives." 162.

In opposition to the French writers, Dr. Prout is not disposed to lay any great stress on *diet* in a case of phosphatic diathesis. He considers the quiet of mind of the patient of far more consequence, and urges forcibly that

> "Absence from care, the exhilarating air of the country, and such exercises as are consistent with the patient's condition, will, perhaps, more than any thing else, contribute to the cure, particularly in the slighter cases, and when the cause is not local injury." 163.

The next subject which engages the attention of the author is that of calculi, considered in a *mechanical* point of view;—that is to say, with reference to their modes of formation and subsequent increase, the symptoms which they produce as solid foreign bodies in the different urinary passages, and the medical treatment to be adopted when they are lodged in different situations.

We are not much pleased with the laboured theory which the author throws out in page 182, to account for the first formation of renal calculi, whether lithic or mulberry. His idea is that they are thrown out in a plastic *hydrated* state, but we cannot avoid expressing our persuasion that this speculation is unnecessary. It does not appear to us to add any thing to general pathology, and it is clearly inapplicable to practice. After noticing the treatment to be pursued in a case of renal calculus, the author is led to consider the growth of the stone, when in the bladder; and here we were struck by an observation which appears to us very questionable. We are told, (Page 195) that the *laminated* structure of calculi shews that their formation has been interrupted—in other words, that it has taken place at distant intervals; for, adds the author, if a calculus was constantly increasing, its texture should be homogeneous. He afterwards compares the structure of a calculus to that of an onion. Does he suppose that the concentric laminæ, which that bulb exhibits, prove "that they wer eformed at different periods, separated by longer or shorter intervals?"

Whatever may have been the previous nature of the calculus, the *phosphatic diathesis always prevails whenever the patient's general health gives way.* The coats of the bladder then become thickened and diseased, and often, he might have added, disorganization of the kidney itself takes place, and death closes the scene.

The *local* treatment of calculi is nearly the same in all the species, consisting principally in the employment of anodynes. "Hyosciamus is in general to be preferred in the lithic acid diathesis, and opium in the phosphatic." (Page 202.) This is urged in consequence of the author's having noticed, that "opium frequently increases the formation of the lithic acid," (page 177) an observation which we think of great importance, considering his extensive recommendation of this remedy in the diseases of the urinary system.

The *general* treatment depends, of course, upon the nature of the diathesis which is present; and it may be understood from what has been already stated. It is important to bear in mind, that while the urine is in its natural state, *the calculus cannot increase in size.* When, therefore, the diathesis, more particularly the lithic diathesis, has been broken in upon by medicine, the effect is to be kept up by strict attention to diet and regimen, and the patient may live on, scarcely knowing that he has a calculus in the bladder.

In the cure of a calculus composed of the phosphates—

"It should be our object, as in all other affections of this description, to restore the urine as speedily as possible to its natural state. I am sorry, however, to be obliged to confess that I have never been able to accomplish this purpose in a single instance, even after the most fair and persevering trial of almost every remedy that has hitherto been recommended, or that I could devise as likely to effect my purpose. The consequence has been, that I have never been able to procure more than a temporary relief from suffering by the various exhibition of opiates, &c. The operation of lithotomy, therefore, seems to be the only alternative in this form of the disease. If, however, the case is doubtful, or the patient refuses it, or his situation will not admit of the performance of the operation, recourse may be had to the means formerly pointed out when the nature of this diathesis was treated of in detail." 206.

The last chapter contains a number of valuable observations on the influence which periods of life, sex, and climate, exert over calculous complaints; on the mortality attending lithotomy, and the circumstances *of a general nature*, which should incline us to recommend, or to dissuade from, that operation. We have occupied so much space with the author's general views, that we have really no room to spare for the details into which we should be led by a full analysis of this chapter. There are two or three remarks, however, which we noted down as particularly interesting, for which we must endeavour to find a place.

Dr. Prout seems to think (page 211) that a large proportion, perhaps the majority, of lithic acid calculi are formed in *very early life;* and that no urgent symptoms are occasioned by them till towards the age of forty.

"The hey-day of life has now past; the individual has perhaps been married for some years, and, from being less active, has become corpulent and gouty; for some time past the irritation in the bladder has been more frequent and urgent, and he observes that his urine is loaded with gravel; he now begins to take the alarm, and to be apprehensive that his complaint may terminate in stone; a surgeon is consulted, who confirms his apprehensions; and here begins the tale of woe; his new disease haunts him perpetually, and soon begins to affect his health and spirits; at length his constitution gives way—an irritable state of the system comes on—the phosphatic diathesis is induced, and a cruel operation, or a miserable death, is the only alternative." 213.

The phosphatic diathesis occurs sometimes as an original disease in the prime of life, without being preceded by the lithic acid, or any other diathesis. (P. 215.) Calculus is less frequent at present than it was formerly. (Page 216.)

The operation of lithotomy is to be recommended without delay, whenever a calculus, no matter of what species, is ascertained to exist in the bladder before puberty—where the phosphatic diathesis is fairly formed—and, lastly, when the urine is loaded with pale-coloured lithate of ammonia; for in this case the phosphatic diathesis cannot be prevented, nor the lithic diathesis restored. On the other hand, the operation may be postponed, when the calculus is small and the lithic diathesis *steadily present*—when the patient is in the prime of life and the health sound, and where he is willing to conform to regular living. Under all other circumstances the retention of the calculus in the bladder is dangerous.

Before parting with Dr. Prout, we must offer him our thanks for the instruction which he has afforded us; and, *for his sake,* express our hopes, that we may have been successful in conveying to our readers a clear and impartial idea of the views which he has formed of the deranged operations of the urinary organs.

